# A comparative multi-objective investigation for metallic and non-metallic materials on the erosion resistance of wind turbine blade with performance evaluation

**DOI:** 10.1038/s41598-025-27700-8

**Published:** 2026-01-06

**Authors:** Ahmed S. A. Abou Taleb, Islam H. Abdelgaliel, Mohamed F. Aly

**Affiliations:** 1 Department of Mechanical Engineering, Faculty of Engineering, Fayoum University, Fayoum, 63514 Egypt; 2 Department of Mechanical Engineering, Al Ryada University for Science and Technology, Al Monoufia, 32897 Egypt; 3 Department of Mechanical Engineering, School of Science and Engineering, The American University in Cairo, AUC Avenue, New Cairo, 11835 Egypt

**Keywords:** Erosion rate, Taguchi array, Wind turbine blade, Angle of attack, Lift-to-drag ratio, Power production, Composites, Mechanical properties, Mechanical engineering, Fluid dynamics

## Abstract

Turbine blade erosion is a significant concern in the power generation industry, as it can reduce efficiency and increase maintenance costs. This study investigates the effect of impact speed, impact angle, and particle size on the erosion rate of turbine blades, using a combination of simulation models and experimental validation. Regression models were developed to analyze the influence of these factors on the erosion rates of both metallic and non-metallic turbine blade materials. The results indicate that erosion rates increase with particle size and impact speed, with impact angle playing a crucial role in the erosion behavior. For metallic alloys, erosion rates decrease with increasing impact angle, whereas, for non-metallic materials, the erosion rate increases proportionally with impact angle. To optimize erosion resistance and aerodynamic performance, three multi-objective optimization algorithms; Multi-objective Genetic Algorithm (MOGA), Multi-objective Pareto Search Algorithm (MOPSA), and Weighted Value Gray Wolf Optimizer (WVGWO), were applied. The optimization process incorporated both the erosion rate and lift-to-drag ratio as objective functions, with the optimal impact angles identified for different materials: 22.5° for Kevlar and fiber glass reinforced epoxy, 28° to 29° for stainless steel, and 32.5° for aluminum alloy. These findings offer valuable insights into the design optimization of wind turbines, aiming to minimize turbine blade erosion and reduce maintenance costs, while improving aerodynamic performance.

## Introduction

Turbine blade erosion is a significant problem in the power generation industry. Erosion can occur when solid particles in the gas stream impact the blades at high velocities, causing material loss and reducing the efficiency of the turbine^1^. The erosion process is complex and influenced by a range of factors, including the speed of the wind, the impact angle (angle of attack) of the particles on the blade surface, and the size and shape of the particles, the concentration of the sand particles in the stream, etc.^2,3^. The effect of these factors on turbine blade erosion has been the subject of extensive research. Several studies have shown that erosion rates increase with increasing speed, as the kinetic energy of the particles increases, leading to more significant impacts and material loss. The angle of attack of the particles on the blade surface also has a significant effect on erosion rates, with higher rates observed at angles of attack between 30 and 45°^4^. Particle size is another critical factor, with larger particles leading to higher erosion rates due to their higher kinetic energy. To mitigate the effects of erosion, various techniques have been developed, such as using coatings and surface treatments on the blades or using wind shields^3^. However, these techniques are not always effective and can increase the cost and complexity of maintenance. Therefore, it is essential to understand the fundamental factors that influence erosion rates to develop more effective strategies to reduce the risk of turbine blade erosion.

**Fig. 1:**
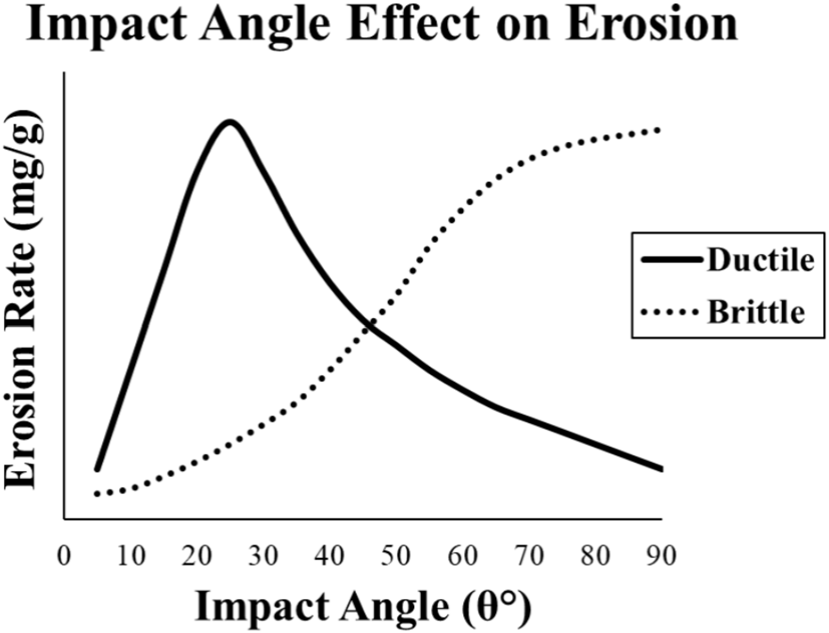
Ductile versus brittle material erosion rate at different impact angles.

Not only the wind kinetics and surrounding environment characteristics are the main factors affecting the erosion rate, but also the wind blade material properties can be effective. A comparative study was carried out to investigate the different erosion rates of different materials under the same conditions but different impact angles. It is found that ductile materials attain higher erosion rates at small acute impact angles in the range between 10 to 30°. On the other hand, brittle materials have the maximum erosion rates at 90° approximately^5^. The same comparison is carried out by Srivastava and Suhane^6^ between aluminum alloy and glass. The erosion rate behavior of ductile and brittle materials against the impact angle can be shown on Fig. 1, while the y-axis is unlabeled for illustration only. Ahamed et al.^7^ proposed erosion maps for different glass fiber reinforced polymer (GFRP) grades as materials for tidal turbines that depict the erosion rates as bell curves with a maximum value at impact angle of 60°. In addition, similar erosion maps were proposed by Kaundal et al.^8^, however, the authors added the fiber loading (10–50 wt. %) as an additional factor that affect the erosion rate. The findings of this study show that composites with fiber loading of 30 wt. % attain the optimal erosion resistance whereas the 50 wt. % loading had the maximum erosion rate. Moreover, the erosion rate of plasma sprayed nickel–aluminum composite is found to be at maximum values between 15 and 45° of impact angle at different speed levels. After 45° onwards, the erosion rate is decreasing gradually. For the effect of speed of erodent particles, it is found that the erosion rate is increasing proportionally with the impact speed^9^. Another important remark is that the ductility or brittleness affects the erosion mechanism greatly. Also, some materials can shift between ductile to brittle performance with proper surface coating. A study uncovered that application of WC-12Co and WC-10Co–4Cr coatings to EN8 steel shifts its wear behavior from ductile to brittle^10^.

As a comparison between metallic alloys (stainless steel and aluminum), Malik et al.^11^ investigated the effect of impact angle and impact velocity on erosion rate for four different materials (AISI 310S, AISI 316, AISI 1020 and Al-6060) using Oka et al.^12^ model as a comparison to the experimental results. First, the interaction between the two parameters had no significance on the erosion rate, hence, they were independent to each other whereas it is confirmed by Oka et al.^12^. Also, the materials showed ductile behavior as shown in Fig. 1. Finally, the hardness of materials had the most influential effect on erosion rate as Al-6060 attained the best erosion resistance upon all comparisons carried out in this study. Meanwhile, Kevlar material is considered a brittle material in nature^13^. Moreover, a comparison study between coated stainless steel and polytetrafluoroethylene (PTFE) focused on optimizing cavitation erosion resistance by analyzing factors like jet velocity, impingement angle, and stand-off distance. The results show that a 90° impingement angle leads to the most erosion, while a larger stand-off distance reduces mass loss. SEM analysis revealed different erosion patterns for each material: SS316 showed plastic deformation, VC + TiC had crater formation, and PTFE showed torn splats. The findings emphasize the importance of adjusting these parameters to improve erosion resistance, with potential applications in reducing cavitation damage^14^.

Another study stated that flexural strength degradation can also be explained by an increase in brittleness of glass fiber reinforced epoxy composites after heat treatment due to cross-linking and chain scission of the composite’s initial molecular structure and a decrease in the inter-facial adhesion force between glass fiber and epoxy caused by oxidation of the epoxy matrix or a difference in the glass fiber and epoxy coefficient of thermal expansions^15^. Therefore, Kevlar and fiber glass reinforced epoxy behave as brittle materials.

Furthermore, a study investigated the surface characteristics, elemental composition, and cavitation erosion resistance of AlN coatings deposited on an aluminum substrate via RF sputtering in argon. Results showed non-homogeneous deposition with minimal porosity, enhanced hydrophobic behavior, and increased erosion resistance compared to the untreated substrate. Optimal cavitation erosion conditions were identified, and XPS analysis revealed key elements such as Al, Na, Cl, O, and N. Regression models demonstrated strong correlations between experimental and predicted results, with the AlN coating reducing erosion by approximately 1.5 times compared to the uncoated substrate^16^. Regarding the coating of stainless steel 316, a study analyzed the morphology, mechanical properties, erosion resistance, and machine learning modeling of plasma-sprayed Si_3_N_4_ + TiC + VC and CrNi-based ceramic coatings. The coatings showed strong mechanical bonding with the substrate, with Si_3_N_4_ + TiC + VC exhibiting pores. Microhardness ranged from 1933 HV (Si_3_N_4_ + TiC + VC) to 499 HV (CrNi). Slurry erosion tests revealed superior erosion resistance for Si_3_N_4_ + TiC + VC at a 90° impingement angle. Over 20 machine learning models were assessed, with Gaussian Process Regressor and ANN (20-layer) showing good predictive performance for mass loss. The study emphasizes the superior erosion resistance of Si_3_N_4_ + TiC + VC coatings, recommending their use to enhance the performance of stainless steel in industrial applications^17^.

The aim of this research is to develop a comparative investigation between different metallic (stainless steel and aluminum) and non-metallic materials (Kevlar and fiber glass reinforced epoxy) on basis of erosion rate and its effecting factor. Also, this work focuses on the multi-objective optimization of effecting factors on the erosion rate and aerodynamic performance. The impact angle (θ), impact velocity (ν) and erodent sand particles (s) are selected as the investigated parameters. Taguchi’s orthogonal array L_9_ is used as a design of experiment method. Using MATLAB, mathematical regression models are developed to simulate the erosion rate behavior of each material. Finally, three multi-objective algorithms are used to find the optimal impact angle that enhances the wear resistance and improves the lift-to-drag coefficient corresponding to the wind turbine performance.

## Materials and methods

In this work, four different materials are selected for wind turbine blades to be investigated and compared according to their erosion rates. The test rig and measuring devices are presented. Finally, the design of experiment is illustrated in detail showing the selected test parameters and their levels.

### Materials

**Fig. 2:**
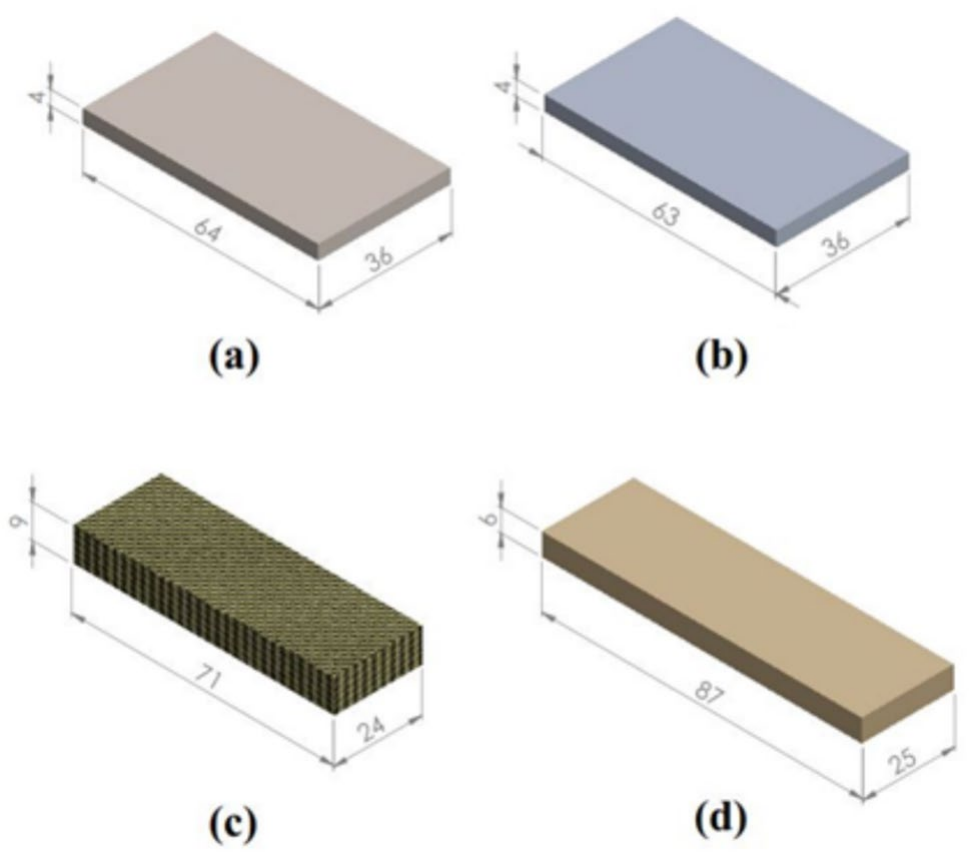
Samples’ geometries (**a**) Stainless steel, (**b**) Al-Alloy, (**c**) Kevlar and (**d**) Fiber glass reinforced epoxy.

The experiment is carried out on four materials: Stainless steel, Al-Alloy, Kevlar and fiber glass reinforced epoxy. The samples’ geometries for each material are shown in Fig. 2.

### Equipment setup

**Fig. 3:**
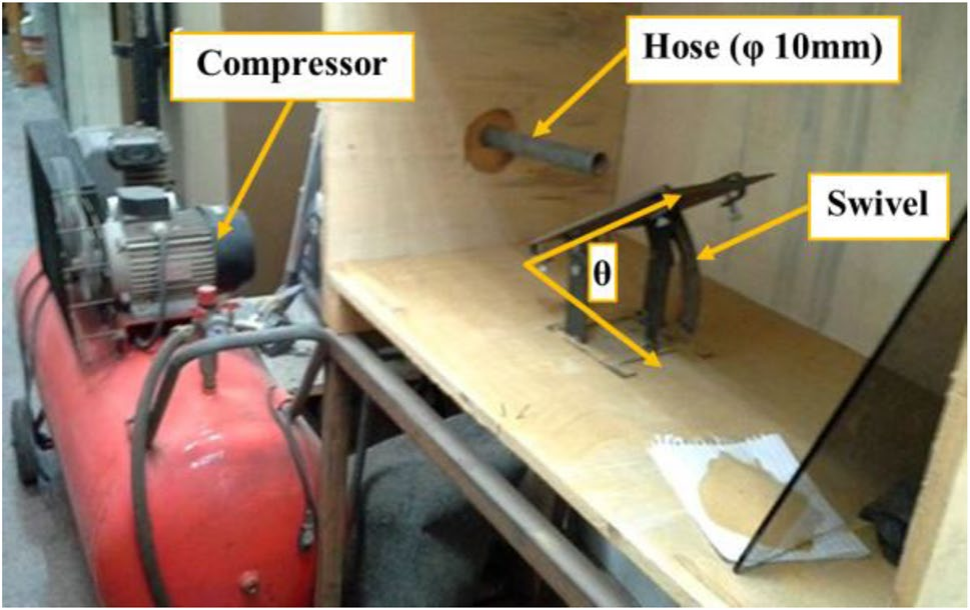
Test rig apparatus.

**Fig. 4:**
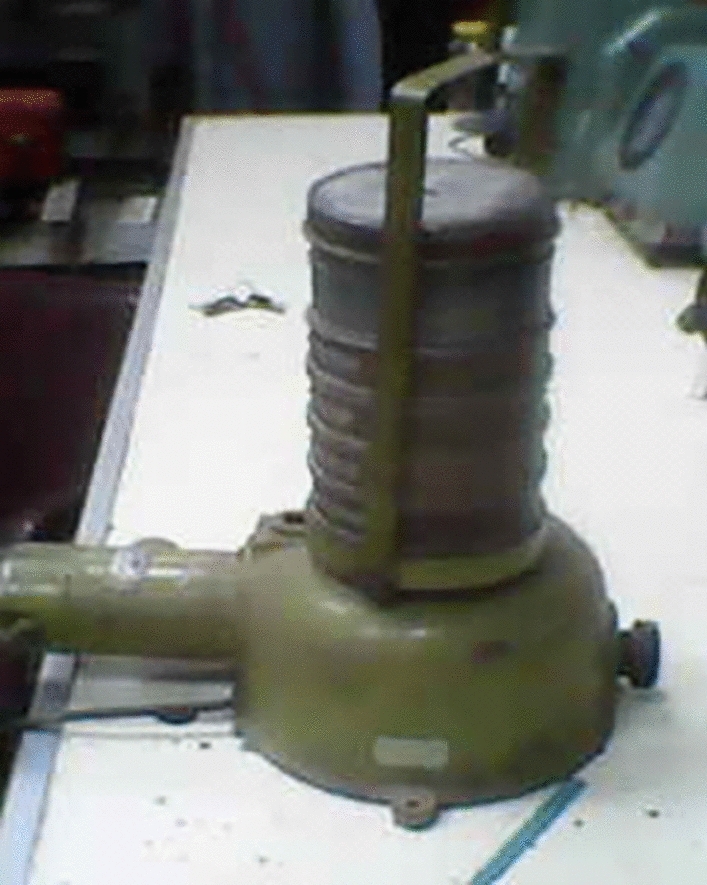
The sieves shaker device (strainer).

The test rig device is custom-made as shown in Fig. 3. The samples are mounted on the swivel to control the variable angles of impact. The used compressor has specs of power 2 hp, pressure 0–12 bars, capacity of 100 L and a hose of 10 mm diameter. The sieves device shaker (strainers), to determine the variable sand sizes, is shown in Fig. 4. The sample weight measuring device is Sartorius digital scale with ± 0.001 g accuracy (Göttingen, Germany). Finally, a digital anemometer AM-4836V (Guangzhou Landtek Instruments Co., Ltd, China) is used to measure the wind speed (impact velocity), in addition to calculating the corresponding air flow rate in m^3^/min.

### Design of experiment

**Fig. 5:**
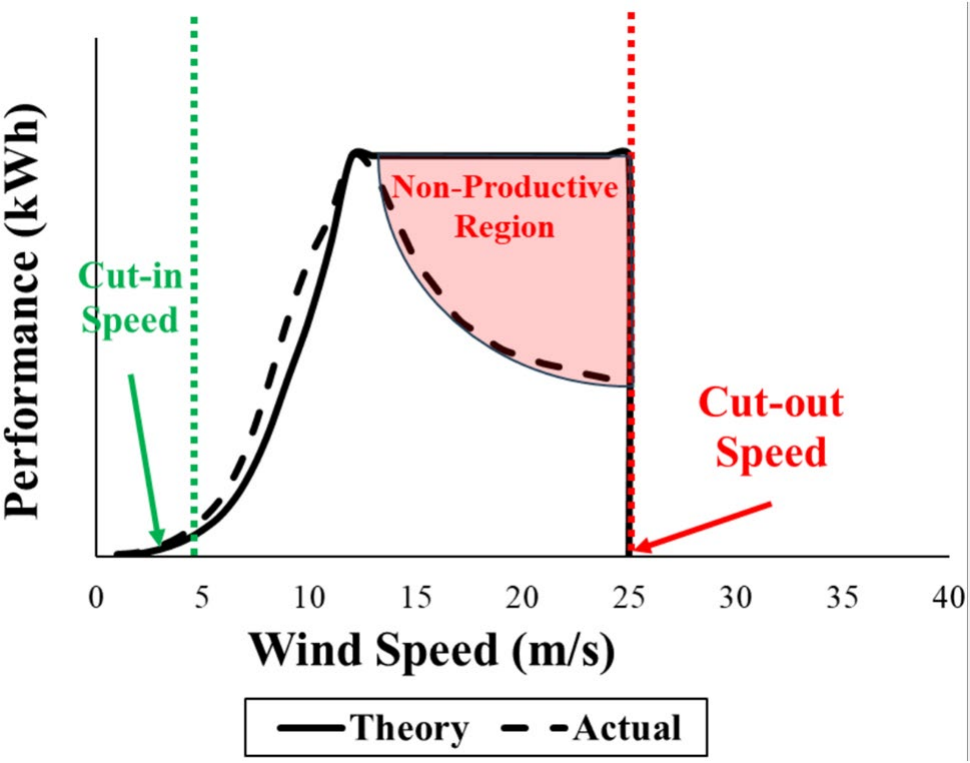
Wind speed vs performance of wind turbine theory.

Three selected testing parameters are used: the impact angle (θ) in °, the impact velocity (*ν*) in m/s and sand size (s) in mm. Each parameter has three levels as shown in Table 1. The reason behind selecting these levels is due to the performance related issues and equipment depreciation factors. For the impact angle (θ), the best practical selection is between the 20 and 50 degrees to keep the lift-to-drag coefficients ratio in a productive range. Meanwhile, Mitchell et al.^18^ revealed that between 8° to 30°, the angle of attack is dependent on Reynolds number (Re), in addition to confirming that nearly 20° to 50° of angle of attack is a useful region as the lift coefficient (C_L_) remains greater than the drag coefficient (C_D_). However, according to the flat plate theory, a study showed that the break-even point between C_L_ and C_D_ is at angle of attack that equal 45° after that the C_L_ goes below the C_D_ value which is undesirable^19^. For the impact velocity (ν), many researchers uncovered the fact of keeping the turbine operating after reaching 12 m/s of speed might depreciate the internal equipment of the turbine (mechanical and electrical)^20,21^. Hence, after a certain wind speed the turbine must be shut down. Hence, the levels of the impact velocity are selected to start from the cut-in speed (approximately 6 m/s) to the peak of rated performance at velocity of 12 m/s as shown in Fig. 5. Neglecting the non-productive region, the white region under the non-productive region is considered in Fig. 5. From the peak of rated performance until the cut-out speed, the efficiency of the turbine decreases in addition to affecting the lifetime of the turbine equipment.

**Table 1:** Testing parameters levels.

Parameters	Unit	Levels		
		1	2	3
Impact angle (θ)	Deg. (°)	22.5	30	45
Impact velocity (*ν*)	m/s	6	9	12
Sand size (s)	mm	0.26	0.365	0.51

The design of experiment is carried out using Taguchi approach with orthogonal array L_9_ that has degrees of freedom (DOF) equal to 8. This means each material is tested through 9 runs as shown in Table 2. Each run is conducted through 3 trials, and the average results are considered.

**Table 2:** Experimental trials following Taguchi’s orthogonal array L_9_.

Run #	Parameter		
	θ (°)	*ν* (m/s)	s (mm)
1	22.5	6	0.26
2	22.5	9	0.365
3	22.5	12	0.51
4	30	6	0.365
5	30	9	0.51
6	30	12	0.26
7	45	6	0.51
8	45	9	0.26
9	45	12	0.365

## Results and discussion

### Experimental results

**Fig. 6:**
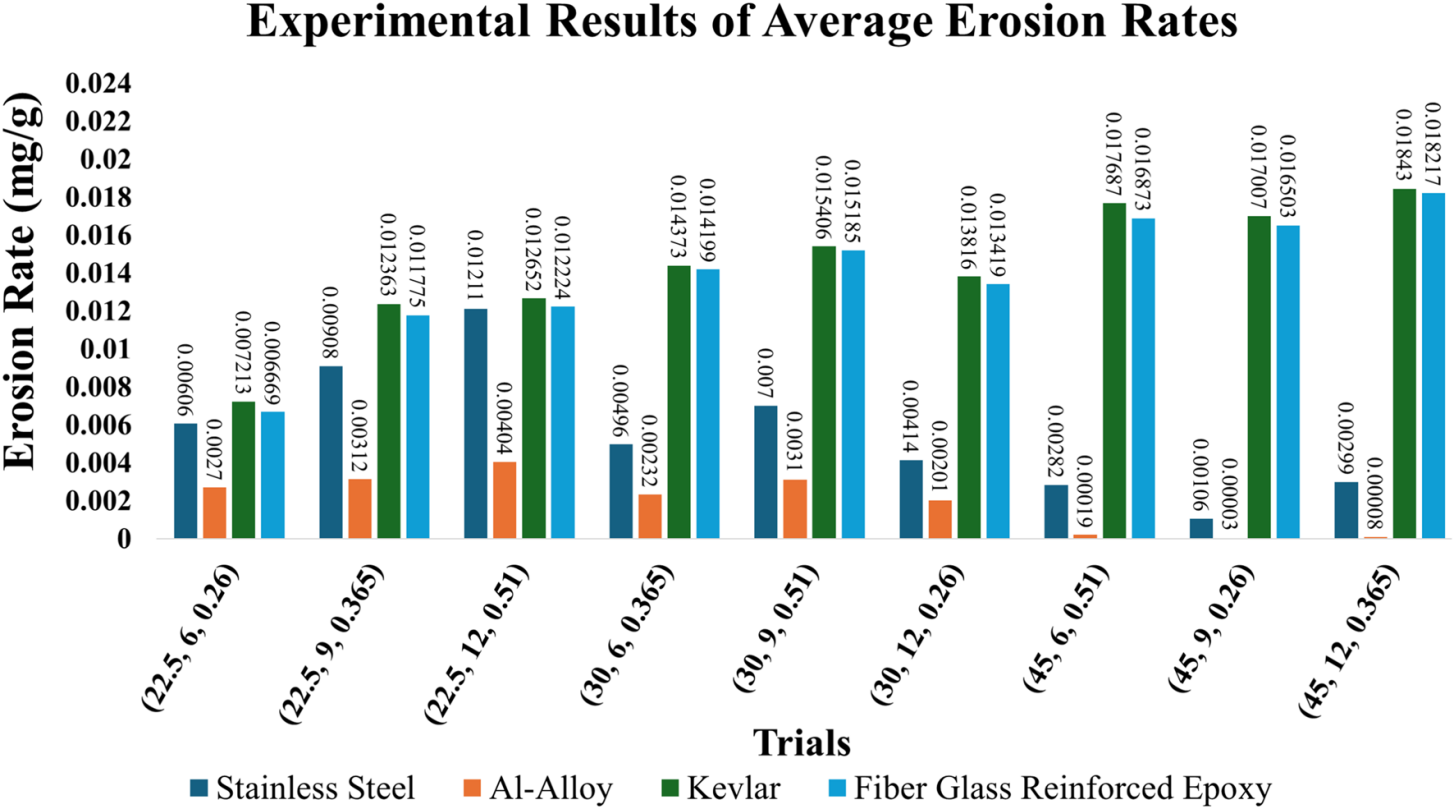
Experimental results of average erosion rates of the used materials.

The addressed response is the erosion wear rate (Ɛ) of the material substrate. This response is calculated by measuring the material loss under experiment in (mg) divided by the amount of sand used in g. The calculation formula as in Eq. (1).

1 $$\varepsilon = \frac{{w_{i} - w_{f} }}{{Q_{P} }}$$

where, Ɛ is the erosion wear rate in mg/g, ${\varvec{w}}_{{\varvec{i}}}$ is the initial sample weight before testing in mg, ${\varvec{w}}_{{\varvec{f}}}$ the final sample weight after testing in mg, and ${\varvec{Q}}_{{\varvec{P}}}$ is the amount of used sand during the test in g. Figure 6 shows the average erosion rates results, on y-axis, of the different used materials out of the experimental trials with the parameters labelled on the x-axis. In addition, Table 3 illustrates the standard deviations and S/N ratios, corresponding to each test trial parameters, of the four tested materials.

**Table 3:** Standard deviations and S/N ratios of the experimental trials of the tested materials.

Trial	Parameters (θ, *ν*, s)	Standard Deviation				S/N ratio			
		SS	Al	Kevlar	FGRE	SS	Al	Kevlar	FGRE
1	(22.5, 6, 0.26)	0.000002	0.000040	0.000479	0.000270	44.35	51.39	42.82	43.51
2	(22.5, 9, 0.365)	0.000015	0.000150	0.000328	0.000349	40.84	50.10	38.16	38.58
3	(22.5, 12, 0.51)	0.000015	0.000080	0.000595	0.000083	38.34	47.87	37.95	38.26
4	(30, 6, 0.365)	0.000099	0.000190	0.000224	0.000369	46.09	52.66	36.85	36.95
5	(30, 9, 0.51)	0.000048	0.000180	0.000119	0.000026	43.10	50.16	36.25	36.37
6	(30, 12, 0.26)	0.000153	0.000170	0.000306	0.000075	47.65	53.91	37.19	37.45
7	(45, 6, 0.51)	0.000116	0.000030	0.000543	0.000100	50.98	74.34	35.04	35.46
8	(45, 9, 0.26)	0.000129	0.000010	0.000416	0.000016	59.46	91.10	35.39	35.65
9	(45, 12, 0.365)	0.000050	0.000020	0.000071	0.000022	50.50	82.12	34.69	34.79

The next step is to develop a mathematical model that fits the testing parameter to the erosion wear rate response for each used material to the variation in the testing parameters. Noting that, the testing parameters are normalized from [−1,1] by Eq. (2) to obtain a higher regression accuracy.

2 $$x_{n} = 2\frac{{x - x_{min} }}{{x_{max} - x_{min} }} - 1$$

The parameters are given a subscript “n” referring to the normalized parameters. This means the parameters in Table 1 are modified as in Table 4.

**Table 4:** Normalized testing parameters.

Parameters	Unit	Levels		
		1	2	3
Impact angle (${{\varvec{\uptheta}}}_{{\mathbf{n}}}$)	Deg. (°)	−1	−0.33	1
Impact velocity (${{\varvec{\upnu}}}_{{\mathbf{n}}}$)	m/s	−1	0	1
Sand size (${\mathbf{s}}_{{\mathbf{n}}}$)	mm	−1	−0.16	1

### Mathematical model regression

Using MATLAB regression learner toolbox beside Minitab software, the regression models of erosion rates for each material model are developed with promising good fit. Also, the prediction of the full factorial design is obtained as the models will be compared to the experimental results. The full factorial design with normalized parameters is created using the parameters’ levels in Table 4.

#### Stainless steel model

**Fig. 7:**
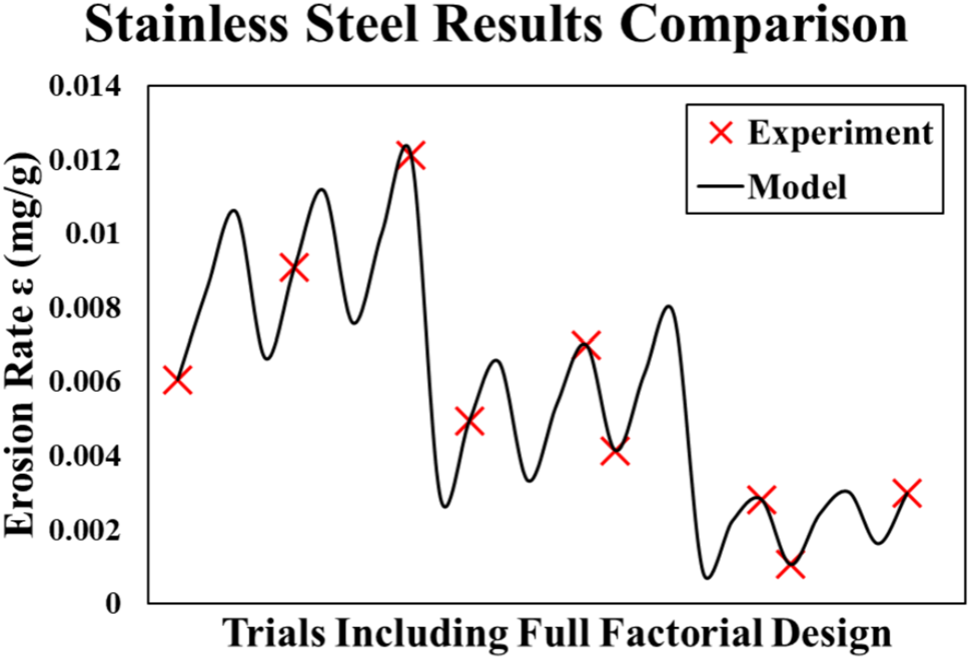
Stainless steel Alloy results (Experiment vs. Model).

The obtained regression model of stainless steel is represented by Eq. (3). This model is extracted from the Minitab regression toolbox. The R-squared of the model is 99.8%. Comparing the experimental results to the model results, it is found that the model can be expanded to include the unexperimented full factorial values from Taguchi orthogonal array L_9_. This comparison is depicted in Fig. 7.

3 $$\begin{aligned} \varepsilon_{SS} = & 0.004393 - 0.003431 \theta_{n} + 0.00057 \nu_{n} + 0.001622 s_{n} + 0.001633 \theta_{n}^{2} \\ & + 0.000183 \nu_{n}^{2} - 0.000556 s_{n}^{2} - 0.000191 \theta_{n} \nu_{n} - 0.000642 \theta_{n} s_{n} \\ \end{aligned}$$

**Fig. 8:**
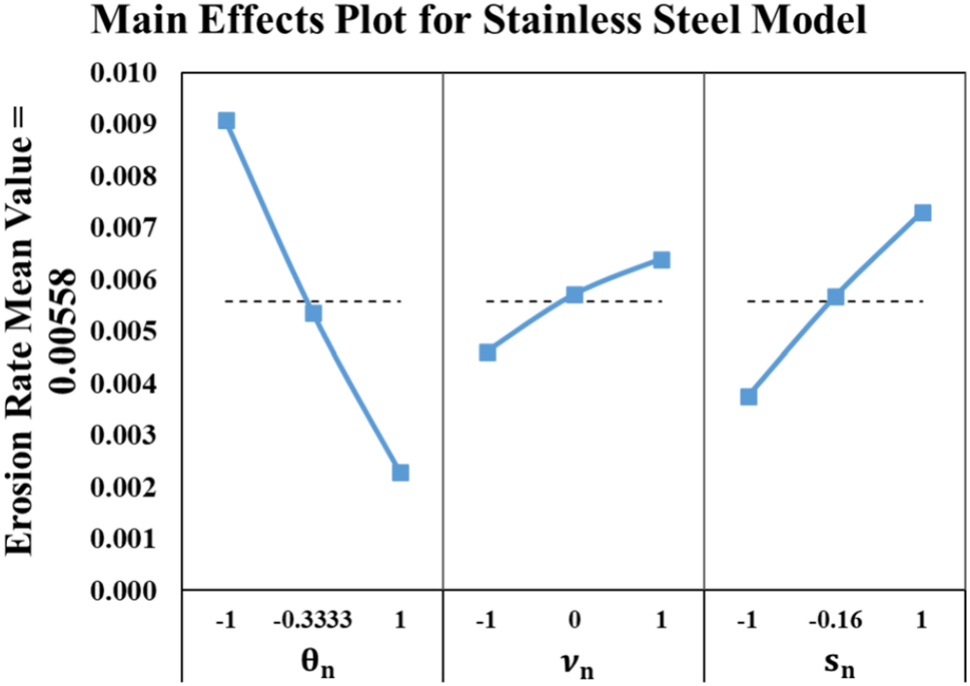
Main effects plot for stainless steel model.

Figure 7 shows the full factorial predicted values by the regression model superimposed on the experimental results of Taguchi’s array. Moreover, the figure shows that the erosion wear rate of stainless steel seems to be direct proportion with sand size (s) and impact velocity (ν) and indirect proportion with impact angle (θ). Hence, as long as the sand particles sizes are smaller, the wind velocity is low and the angle of attack on the wind turbine blade is nearly making the wind stream parallel to blade surface, this leads to the most desirable wear rate for the stainless-steel alloy. The main effects plot of the testing parameters on the erosion rate mean value is illustrated in Fig. 8. This plot indicates that θ is the most influential factor affecting the erosion rate of the stainless steel followed by s and v in the order mentioned.

##### Al-Alloy Model

In this model, the MATLAB stepwise quadratic regression toolbox is used. The obtained model is presented in Eq. (4). The *p*-value of the model is 0.00596 while the R-squared and R-adjusted values are 100% both.

4 $$\begin{aligned} \varepsilon_{Al} = & 0.0023248 - 0.0013969 \theta_{n} + 0.00015741 \nu_{n} + 0.00036498 s_{n} \\ & - 0.00053519 \theta_{n}^{2} - 0.00019293 \nu_{n}^{2} - 0.00015476 \theta_{n} \nu_{n} + 0.00037244 \nu_{n} s_{n} \\ \end{aligned}$$

**Fig. 9:**
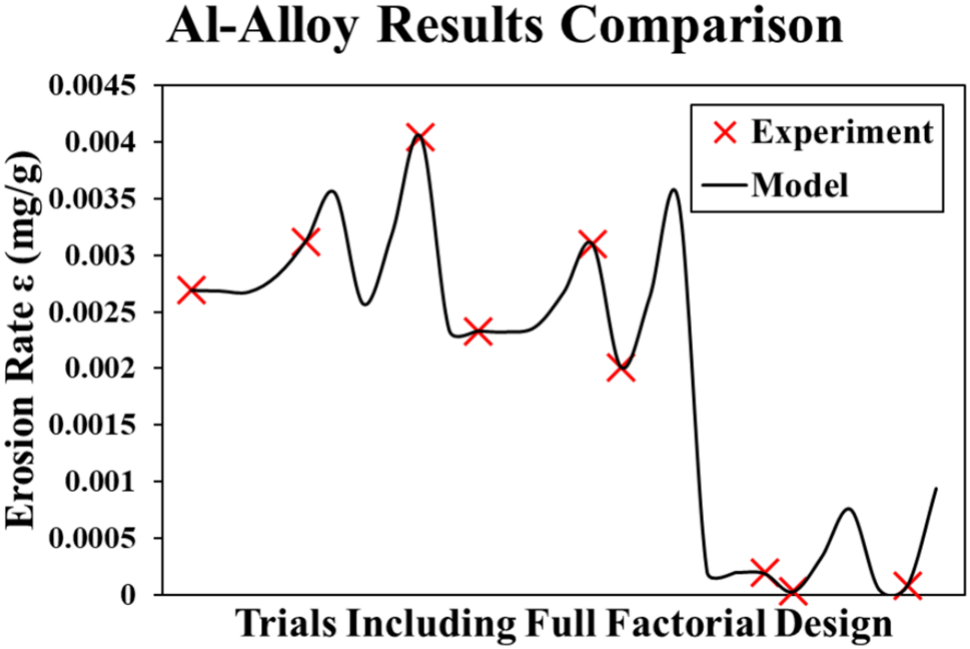
Al-Alloy results (Experiment vs. Model).

Again, the comparison between the experimental and the model results, including the full factorial design of the experiment, is illustrated in Fig. 9. The most effective parameter on the erosion rate of the Al-Alloy is the impact angle (θ). The effect of the impact speed is negligible while the sand size comes in the second place as an influential parameter.

**Fig. 10:**
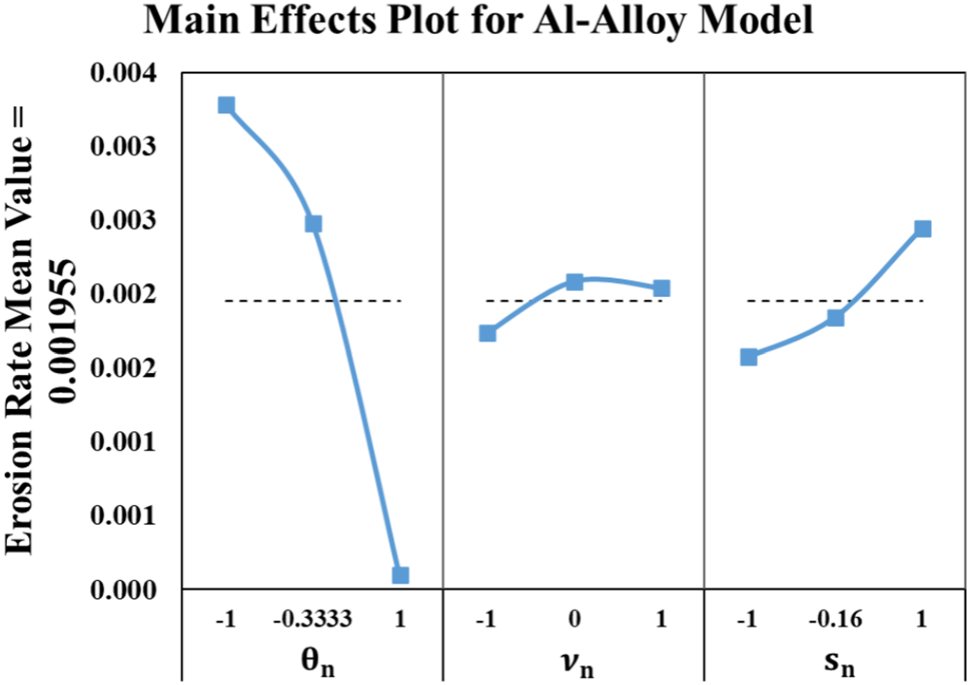
Main effects plot for Al-Alloy model.

Figure 10 shows the main effects plot of testing parameters on the erosion rate of the Al-Alloy. Similar to the stainless-steel alloy, increasing θ with smaller sand sizes (s), while the impact velocity has no significant effect, achieves best wind turbine blade durability.

To wrap up, the metallic materials are greatly affected by the angle of attack (θ) and the sand particle size (s).

##### Kevlar model

**Fig. 11:**
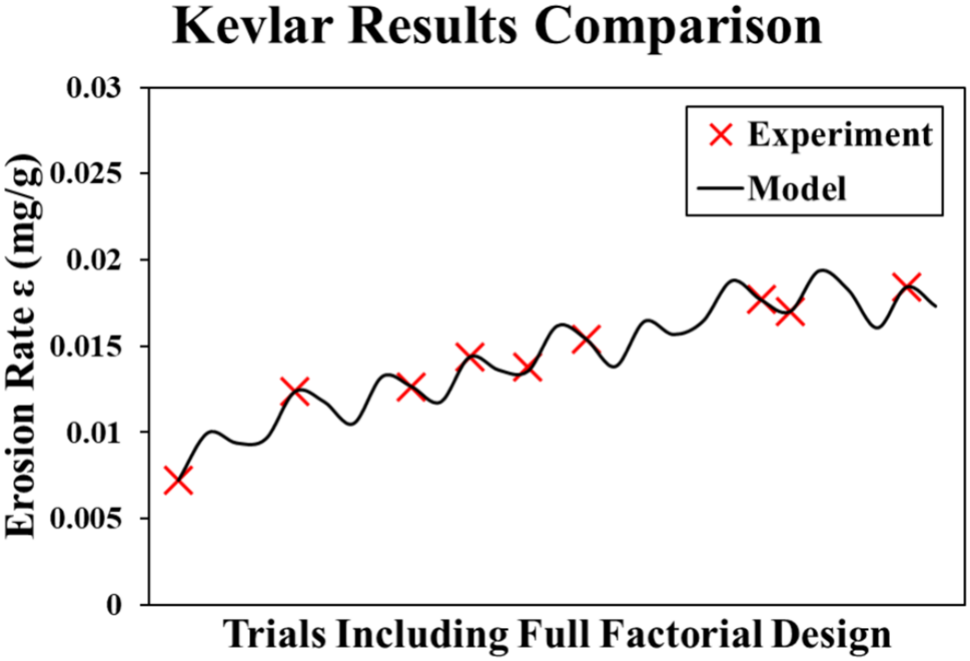
Kevlar results (Experiment vs. Model).

The Kevlar model is developed using MATLAB quadratic regression toolbox as in Eq. (5). This model has R-squared value of 100% as shown in Fig. 11. The experimental results are in perfect match to the model results.

5 $$\begin{aligned} \varepsilon_{Kev} & = 0.01771 + 0.00347 \theta_{n} + 0.000728 \nu_{n} + 0.000855 s_{n} - 0.001651 \theta_{n}^{2} \\ & \quad - 0.000763 \nu_{n}^{2} - 0.0001889 s_{n}^{2} - 0.000921 \theta_{n} \nu_{n} - 0.000225 \theta_{n} s_{n} \\ \end{aligned}$$

**Fig. 12:**
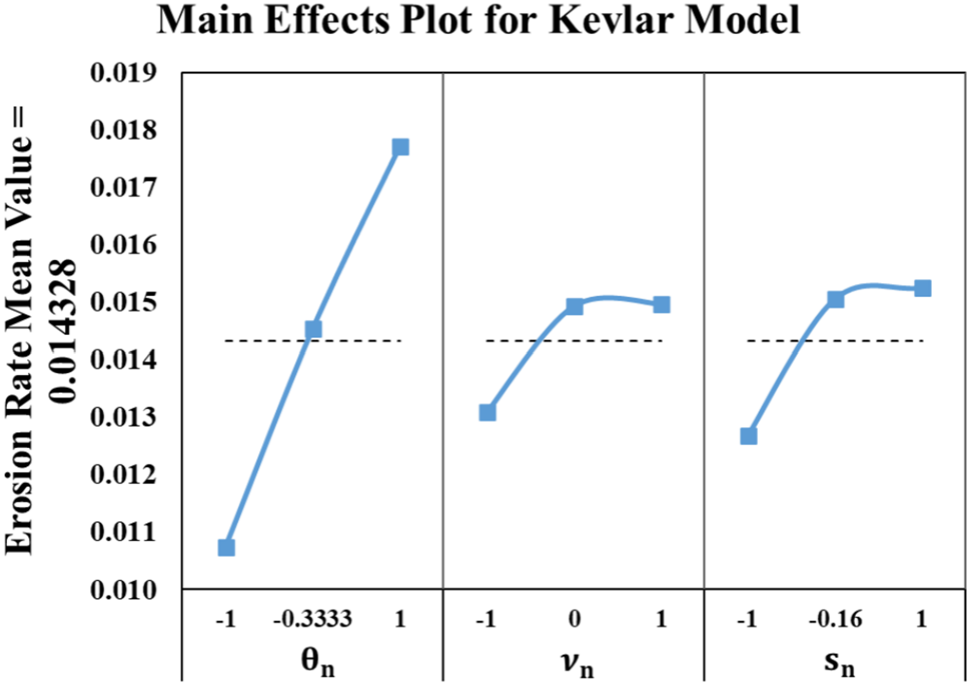
Main effects plot for Kevlar model.

The behavior of the model is like a periodic linear trend because every 9 trials θ is constant and the erosion rate of Kevlar material increases with the increase of the impact angle. This is the reason for the model proportional behavior. Meanwhile, the other two parameters are the reason for the periodic behavior as shown in Fig. 12. As long as the sand particle sizes (s) are close to the upper and lower bounds, the erosion rate decreases. Meanwhile, the velocity is most desirable near the lower bound.

##### Fiber glass reinforced epoxy model

**Fig. 13:**
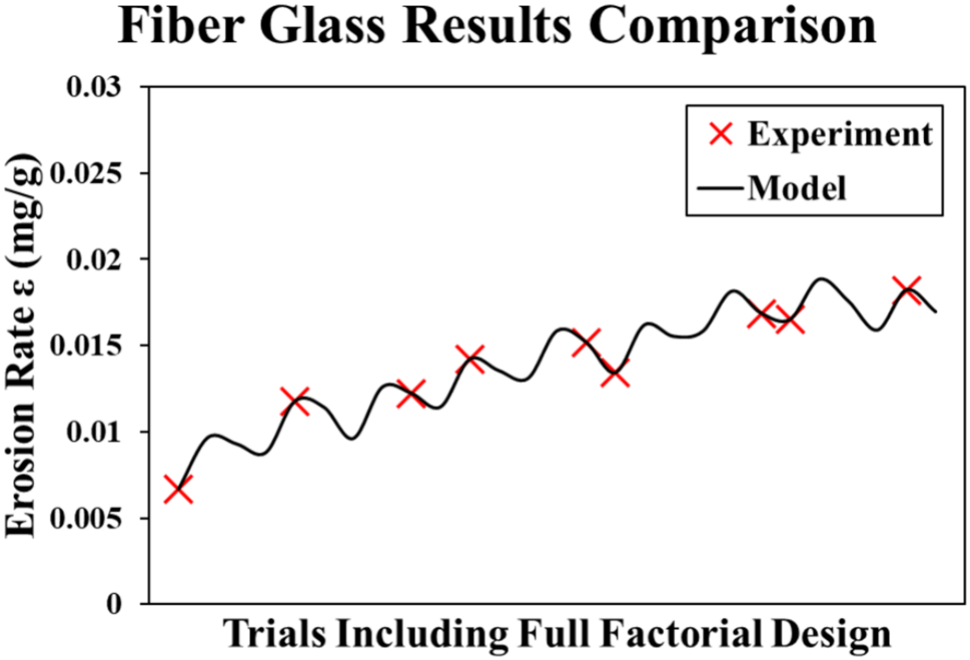
Fiber glass reinforced epoxy results (Experiment vs. Model).

**Fig. 14:**
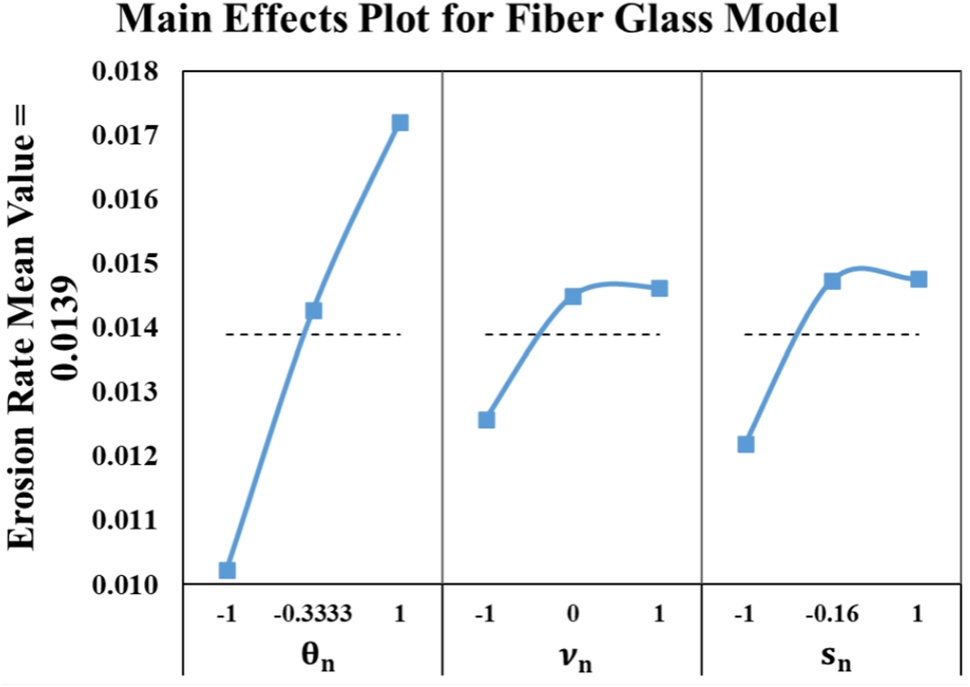
Main effects plot for Fiber glass reinforced epoxy model.

The last material model is obtained by Minitab regression toolbox as shown in Eq. (6). Similar to the Kevlar model, the R-squared value is 100%, and the same model behavior as it is a periodic linear trend for the same reasons mentioned previously. The experiment and model results comparison are presented in Fig. 13. In addition, the main effect plot for fiber glass reinforced epoxy model is depicted in Fig. 14. This behavior is observed to be similar to the study on the glass/epoxy laminates in^22^.

6 $$\begin{aligned} \varepsilon_{Kev} = & 0.01744 + 0.003467 \theta_{n} + 0.000754 \nu_{n} + 0.000921 s_{n} - 0.001935 \theta_{n}^{2} \\ & - 0.000656 \nu_{n}^{2} - 0.001931 s_{n}^{2} - 0.000714 \theta_{n} \nu_{n} - 0.000388 \theta_{n} s_{n} \\ \end{aligned}$$

The most influential factor on the erosion rate of the fiber glass material is the angle of attack (θ). Increasing θ leads to non-desirable wear in the material. Noticeably, the erosion rate of fiber glass materials is the lowest at the lower bounds of each tested parameter.

**Fig. 15:**
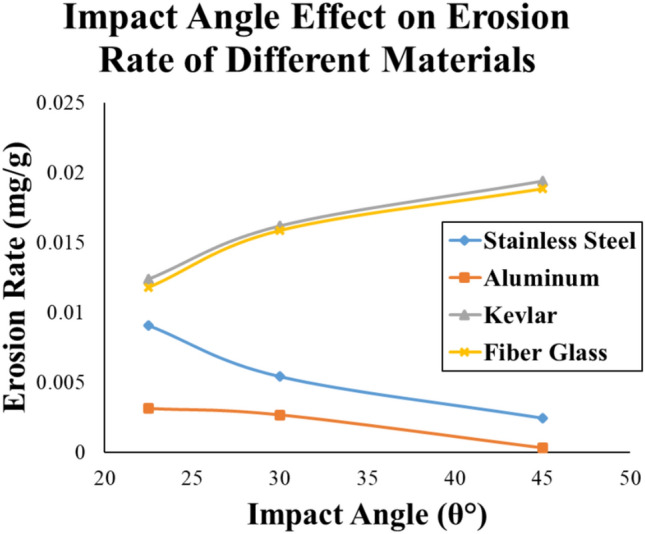
Erosion rate vs impact angle (θ) comparison between all materials at constant ν = 9 m/s and s = 0.365 mm.

Eventually, the most durable condition for all materials occurs when the sand particle sizes (s) are the lowest, the impact velocity is the lowest. As for the impact angle, the highest angle is recommended for aluminum and stainless-steel alloys, whereas the lowest impact angle is preferred for the Kevlar and fiber glass materials. Finally, the Al-Alloy material attains the highest erosion resistance, with erosion rates varies from 0.000028 to 0.004042 mg/g, compared to the other investigated materials as shown in Fig. 15, however, the weight and cost of manufacturing the wind turbine blades are very important considerations to be taken in account.

### Multi-objective optimization results

In order to simplify the model, the sand particles size (s) is uncontrollable factor, hence, considering the maximum sand sizes, for the worst case scenario, (s = 0.51 mm) and substituting in Eqs. (3) to (6) besides constraining the impact velocity (ν) at its maximum velocity (ν = 12 m/s), according to the performance map giving by and as illustrated in Fig. 5, one gets the following erosion rate functions as an impact angle (θ) dependent as follows in Eqs. (7) to (10):

7 $$\varepsilon_{SS} = 0.001633 \theta_{n}^{2} - 0.004264 \theta_{n} + 0.006212$$

8 $$\varepsilon_{Al} = - 0.00053519 \theta_{n}^{2} - 0.00155166 \theta_{n} + 0.0030267$$

9 $$\varepsilon_{Kev} = - 0.001651 \theta_{n}^{2} + 0.002324 \theta_{n} + 0.0183411$$

10 $$\varepsilon_{Kev} = - 0.001935 \theta_{n}^{2} + 0.002365\theta_{n} + 0.016528$$

This places the erosion rate function for each material as the first objective function. The second objective function is derived from the lift-to-drag coefficients ratio C_L_/C_D_ by Mitchell et al.^18^ and Hassanzadeh et al.^19^. Hassanzadeh et al. used the theoretical mathematical model developed by Viterna and Janetzke^23^ in 1982 and applied it on the optimization of the S809 airfoil with aspect ratio (*AR* = 14). This mathematical model is represented in Eqs. (11) and (12).

$$C_{D} = B_{1} \sin^{2} \theta + B_{2} \cos \theta$$

where,

$$B_{1} = C_{D\;\max } = 1.11 + 0.018AR$$

11 $$B_{2} = \frac{{\left( {C_{D\;stall} - C_{D\;\max } \sin^{2} \theta_{stall} } \right)}}{{\cos \theta_{stall} }}$$

$$C_{L} = A_{1} \sin 2\theta + A_{2} \frac{{\cos^{2} \theta }}{\sin \theta }$$

where,

$$A_{1} = \frac{{B_{1} }}{2}$$

12 $$A_{2} = \left( {C_{L\;stall} - C_{D\;\max } \sin \theta_{stall} \cos \theta_{stall} } \right) \times \frac{{\sin \theta_{stall} }}{{\cos^{2} \theta_{stall} }}$$

For typical stall angle, $\theta_{stall} = 15^\circ$, and given that AR = 14. Solving for $A_{2}$ and $B_{2}$ and substituting in Eqs. (11) and (12), one can reduce the previous equation to Eq. (13).

$$C_{D} = 1.362\sin^{2} \theta + 0.00079\cos \theta$$

13 $$C_{L} = 0.681\sin 2\theta + 0.1996\frac{{\cos^{2} \theta }}{\sin \theta }$$

Last, the lift-to-drag ratio can be obtained by the following simplified Eq. (14).

14 $$\frac{{C_{L} }}{{C_{D} }} = \frac{0.681\sin 2\theta + 0.1996\cos \theta \cot \theta }{{1.362\sin^{2} \theta + 0.00079\cos \theta }}$$

Meanwhile, Mitchell’s model is re-developed by Minitab regression, and a 3rd order polynomial with R-squared value of 98.08% is constructed in Eq. (15)

15 $$\frac{{C_{L} }}{{C_{D} }} = 14.80 - 1.030 \theta + 0.02655 \theta^{2} - 0.000233 \theta^{3}$$

**Fig. 16:**
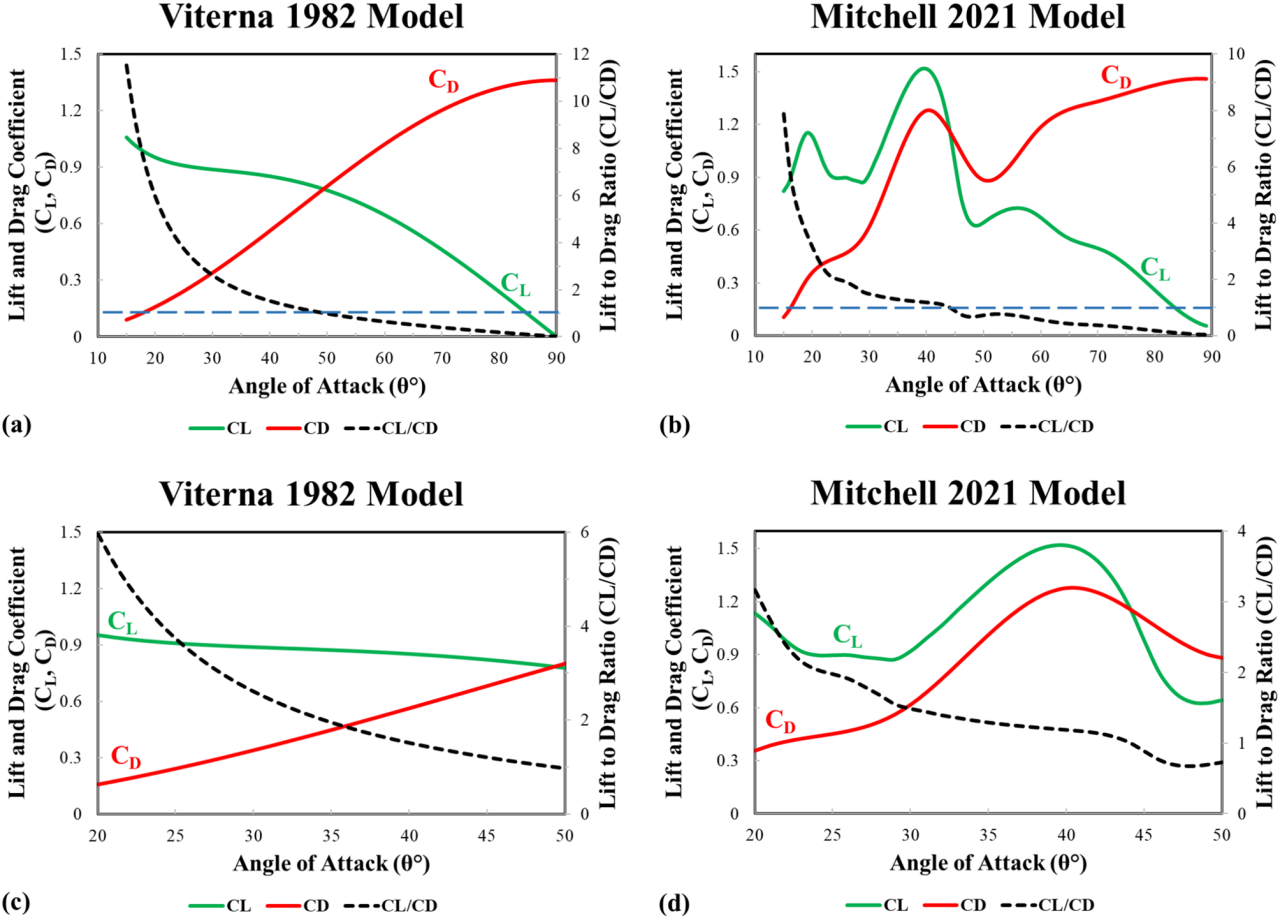
Lift-to-drag coefficients ratio according to Mitchel and Viterna vs angle of attack (**a**) and (**b**) whole model (15° to 90°), (**c**) and (**d**) optimization boundaries (20° to 50°).

Equations (14) and (15) represents the black dashed line in Fig. 16, which are the second objective functions. Figure 16 shows the lift-to-drag ratio comparison between Mitchell and Viterna models. Figure 16a,b represent the complete models derived for the angle of attack from 15° to 90°. To simplify, the plots are reduced to the boundaries of the multi-objective optimization model.

In this stage, the multi-objective optimization model is developed as presented in Table 5. Three multi-objective optimization algorithms are used; (1) multi-objective genetic algorithm (MOGA), (2) multi-objective pareto-search algorithm (MOPSA) and (3) weighted value gray wolf optimizer (WVGWO) by^24,25^.

**Table 5:** Optimization model description.

Item	Description
Number of variables	1
Decision variables	Impact angle (θ)
Linear Inequality	$\left[ {} \right]$
Linear Equality	$\left[ {} \right]$
Lower bounds	22.5°
Upper bounds	45°
Initial point	33.75°, otherwise it depends on the algorithm
Objective functions	Minimize ($\varepsilon_{n}$), for each material model (7) to (10)
	Maximize $\left( {\frac{{{\text{C}}_{L} }}{{{\text{C}}_{D} }}} \right)_{n}$ (13) and (14) or Minimize $\left( {1 - \left( {\frac{{{\text{C}}_{L} }}{{{\text{C}}_{D} }}} \right)_{n} } \right)$

Taking into consideration the normalization of both objective functions’ values from 0 to 1, hence, the subscript *n* indicates the normalized values of the erosion rate and the lift-to-drag ratio. This is to obtain precise optimization results as the two objectives remain on the same scale.

#### *MOGA *model

**Fig. 17:**
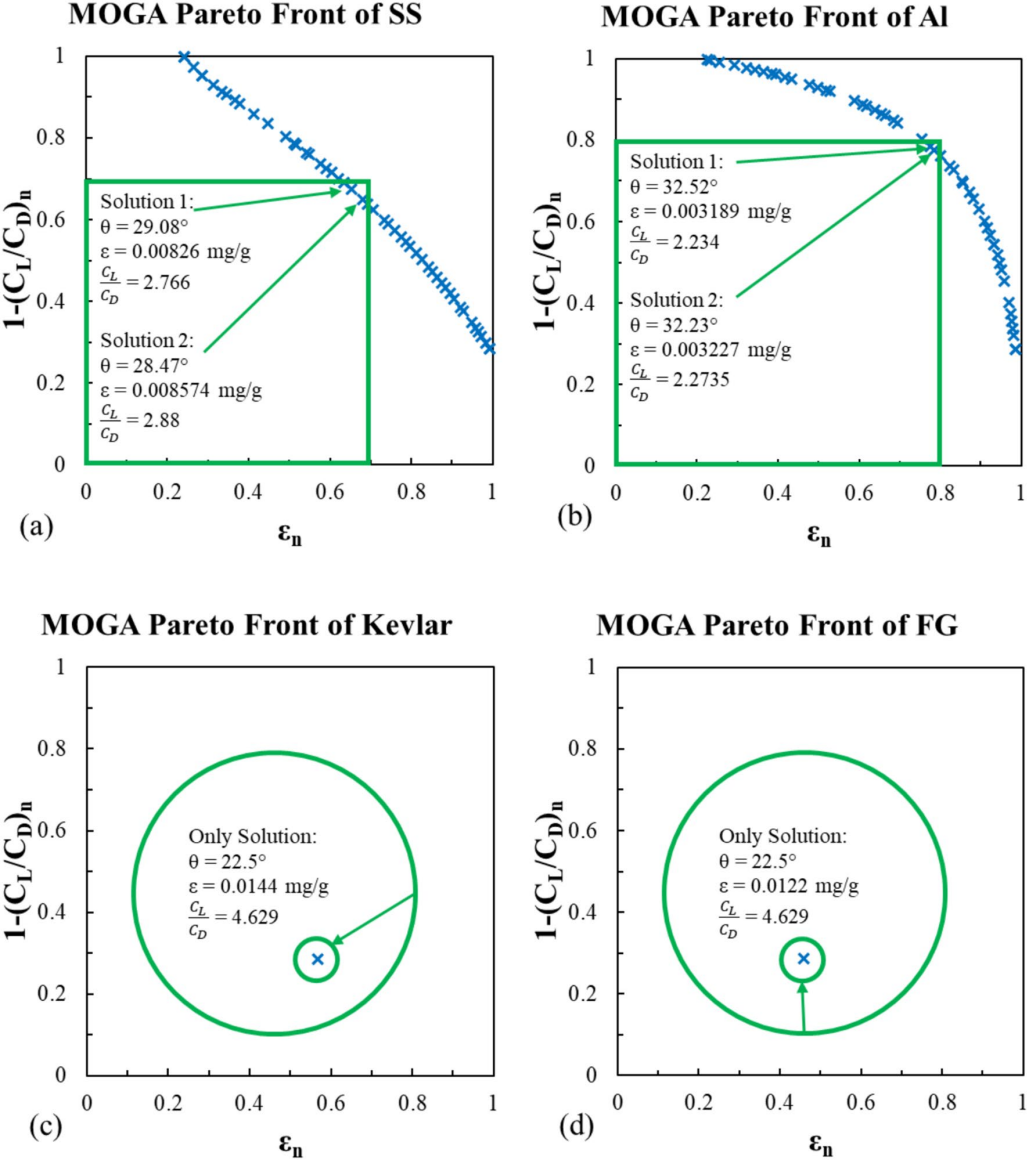
MOGA pareto front for the erosion rate using Viterna model for (**a**) stainless steel, (**b**) Al-Alloy, (**c**) Kevlar and (**d**) fiber glass.

**Fig. 18:**
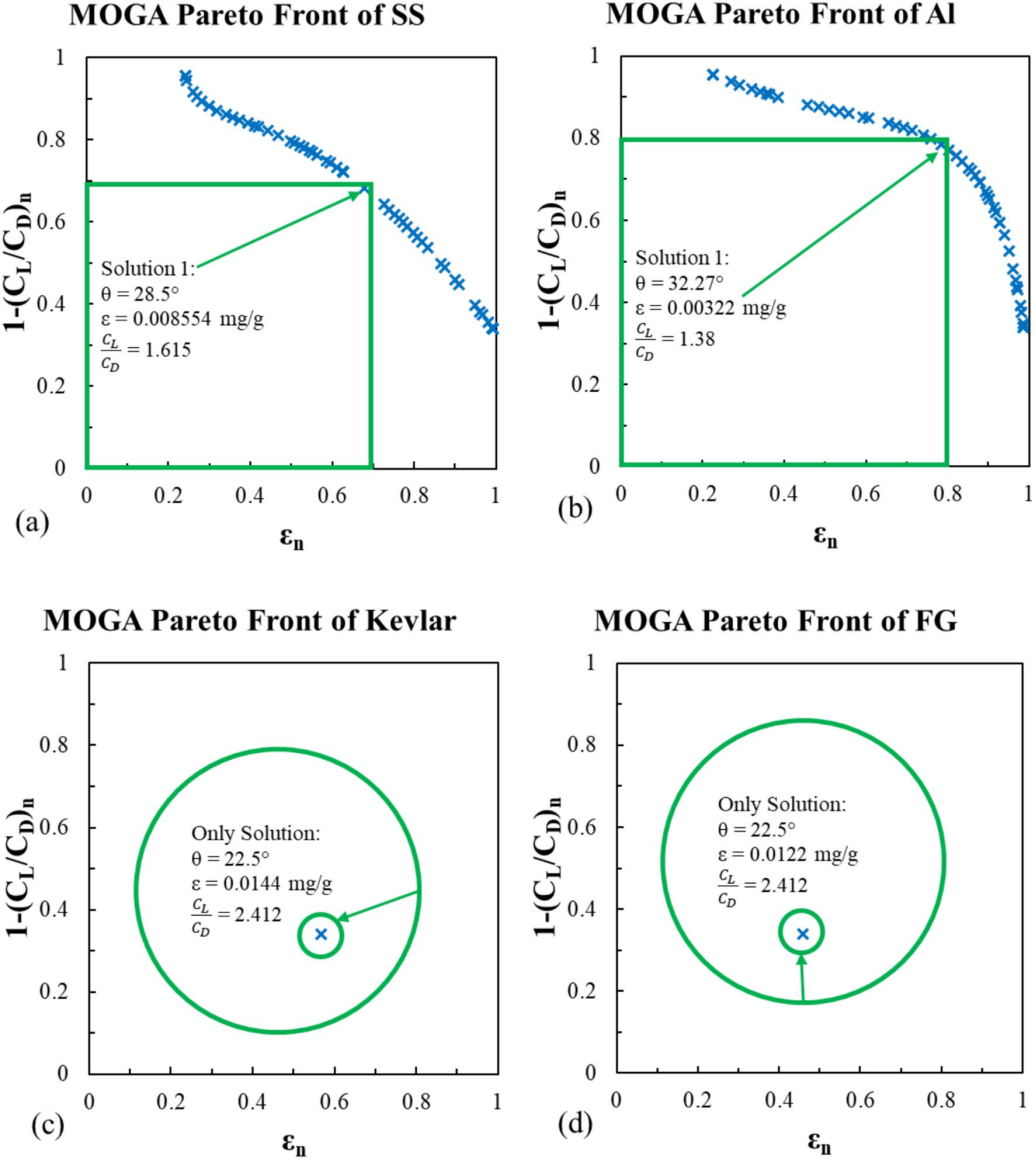
MOGA pareto front for the erosion rate using Mitchell model for (**a**) stainless steel, (**b**) Al-Alloy, (**c**) Kevlar and (**d**) fiber glass.

Using MATLAB optimization toolbox, the MOGA function “gamultiobj” results are presented in Figs. 17 and 18, for Viterna and Mitchell models respectively. The additional options used for the function are maximum stall generations of 50, and the maximum generations are 100. The green squares and circles in these figures indicate the feasible solution areas. For the stainless steel and Al-Alloy, the pareto front of the MOGA results show that there is always a trade-off between the erosion rate and the lift-to-drag ratio. This appears in both Viterna and Mitchell models as shown in Figs. 17a,b, and 18a,b.

The optimal impact angle is between 28.47° and 29.08° for the stainless steel in the Viterna model resulting in an erosion rate of an average of 0.008417 mg/g and an average lift-to-drag ratio of 2.823. Similarly, the optimal impact angle in the Mitchell model is 28.5° with respect erosion rate of 0.0088554 mg/g and lift-to-drag ratio of 1.615.

Meanwhile, the optimal impact angle of the Al-alloy model is 32.3° approximately. This resulted in an average value of erosion rate of 0.003212 mg/g. However, the lift-to-drag ratio is 2.253 on average for the Viterna model and 1.38 for the Mitchell model.

Finally, as discussed in the experimental results section, the impact angle has direct proportional with the erosion rate in cases of Kevlar and fiber glass materials. Hence, the best desirable impact angle corresponding to the best erosion resistance is the lowest. Similarly, the lift-to-drag ratio is at its highest value when the impact angle is the lowest. Therefore, for the Kevlar and the fiber glass models, the optimization process converged directly to a single feasible solution by considering the lower boundary of the impact angle at θ = 22.5°, without revealing the broader feasible solution space. This approach resulted in a specific optimal solution without exploring or illustrating the entire range of possible feasible solutions. The corresponding results are shown in Figs. 17c,d and 18c,d.

#### MOPSA model

**Fig. 19:**
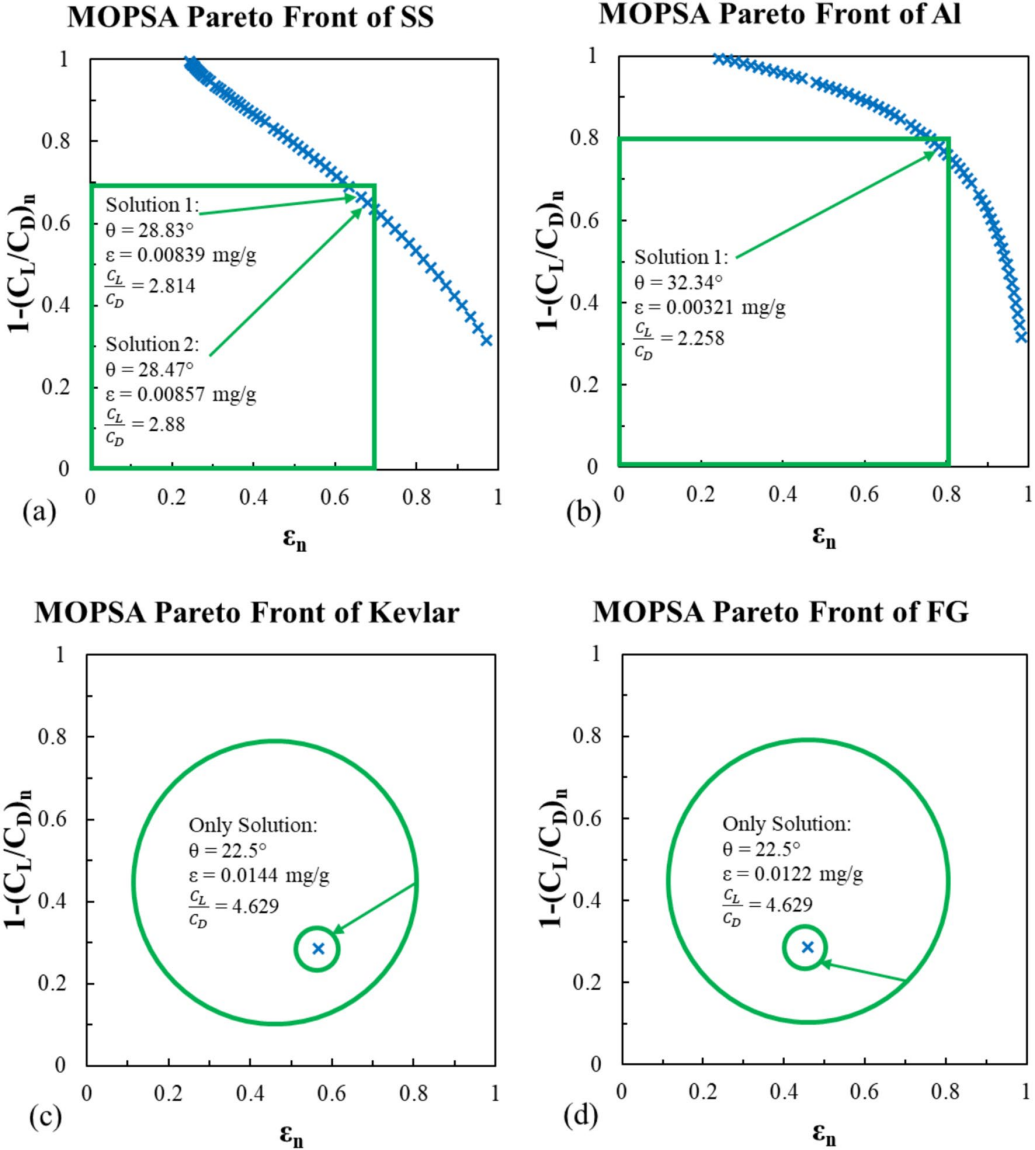
MOPSA pareto front for the erosion rate using Viterna model for (**a**) stainless steel, (**b**) Al-Alloy, (**c**) Kevlar and (**d**) fiber glass.

**Fig. 20:**
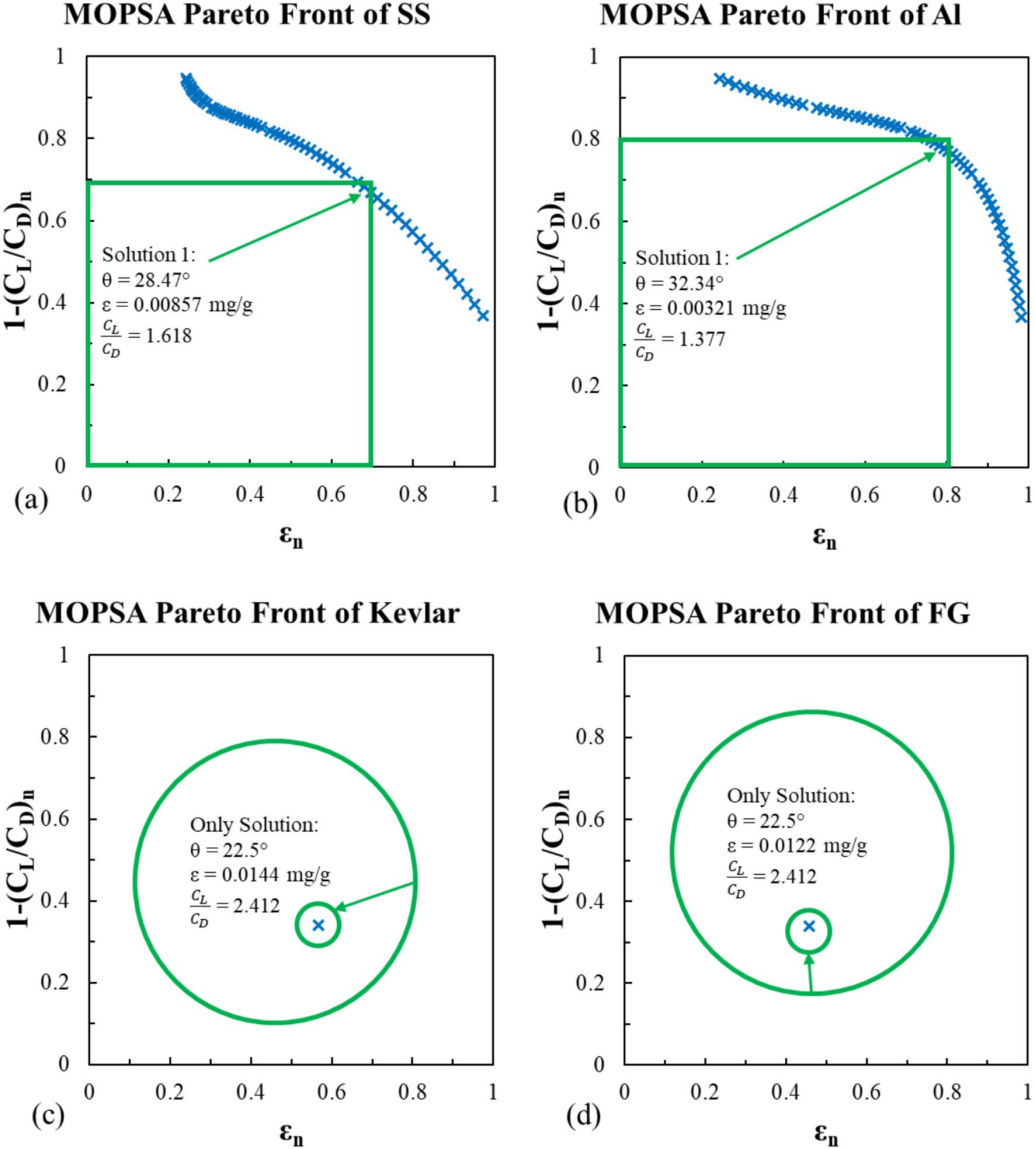
MOPSA pareto front for the erosion rate using Mitchell model for (**a**) stainless steel, (**b**) Al-Alloy, (**c**) Kevlar and (**d**) fiber glass.

Following the same procedure in MOGA model, MOPSA uses iterative method not populations. The additional options are maximum iterations of 100 and the pareto set change tolerance of 1 × 10^–50^. The pareto front of MOPSA results are presented in Figs. 19 and 20. For the stainless steel material, MOPSA results match the MOGA ones as the optimal impact angle is repeated at the value of 28.47° with corresponding erosion rate of 0.00857 mg/g and lift-to-drag ratio of 2.88 as shown in Fig. 19a. In the Al-Alloy case, the optimal impact angle is the same for the Viterna and Mitchell models at 32.34° resulting the same erosion rate of 0.00321 mg/g and different lift-to-drag ratios of 2.258 and 1.377, respectively as shown in Fig. 19b and 20b. Again, the optimal results for the Kevlar and fiber glass materials are at the lower boundary at 22.5° in both lift-to-drag models as shown in Figs. 19c,d, and 20c,d.

#### WVGWO model

**Fig. 21:**
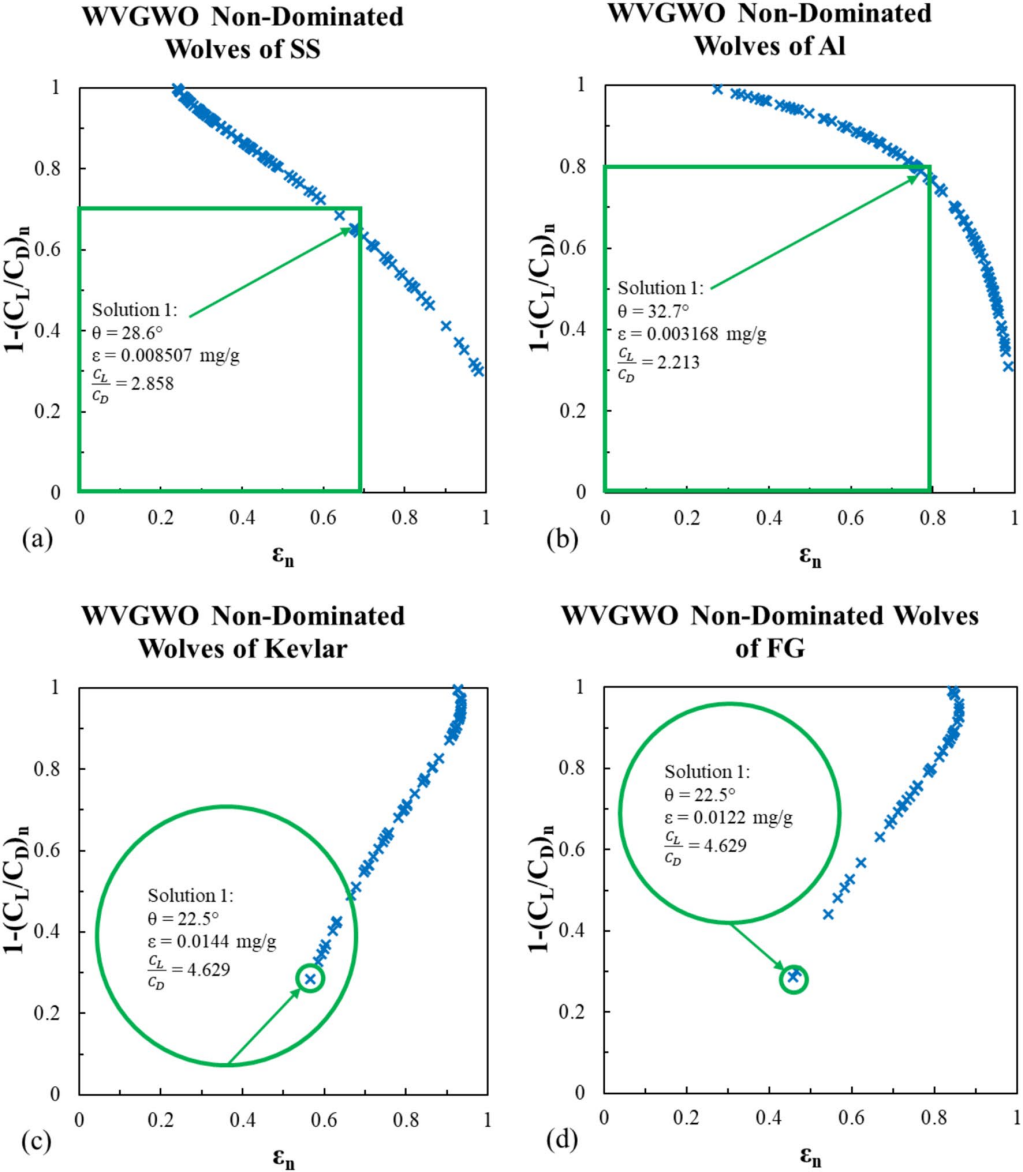
WVGWO non-dominated solutions for the erosion rate using Viterna model for (**a**) stainless steel, (**b**) Al-Alloy, (**c**) Kevlar and (**d**) fiber glass.

**Fig. 22:**
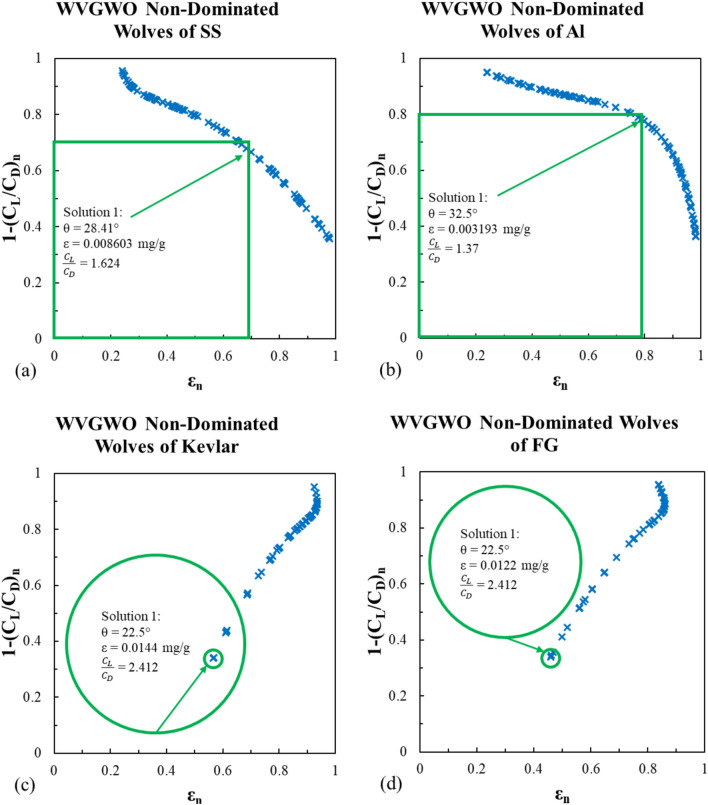
WVGWO non-dominated solutions for the erosion rate using Mitchell model for (**a**) stainless steel, (**b**) Al-Alloy, (**c**) Kevlar and (**d**) fiber glass.

This algorithm is a bio-inspired simulation of the wolf pack’s hunting mission. The pack always has an alpha wolf (leader), lower rank wolves; beta and delta, and the rest of the group is known as omega wolves. The WVGWO starts with an initial wolf pack (population) that is seeking prey (best solutions) and traveling in a specific direction. If the pack finds prey in a specific place (parameters in the current population), the winner wolf advances to a higher rank in the next search. Throughout the search process, these promotions are saved in an archive. The non-dominated wolves are the plotted data on Figs. 21 and 22.

This algorithm provided nearly the same optimal solutions for the stainless steel and Al-Alloy models. For the Kevlar and fiber glass models, it did not converge at the beginning like MOGA and MOPSA at the lowest θ, however, the algorithm strived to find another solution in spite of exceed the feasible solution area, and reversed the non-dominated wolves’ linear trends as shown in Figs. 21c,d, and 22c,d.

To wrap up, the summary of the multi-objective optimization carried out in this research is presented in Table 6.

**Table 6:** Multi-objective optimization results summary.

L/D ratio	Material	MOGA			MOPSA			WVGWO		
		θ	Ε	$\frac{{{\text{C}}_{L} }}{{{\text{C}}_{D} }}$	Θ	ε	$\frac{{{\text{C}}_{L} }}{{{\text{C}}_{D} }}$	θ	ε	$\frac{{{\text{C}}_{L} }}{{{\text{C}}_{D} }}$
Viterna	SS	29.08 28.47	0.00826 0.008574	2.766 2.88	28.83 28.47	0.00839 0.00857	2.814 2.88	28.6	0.008507	2.858
	Al-Alloy	32.52 32.23	0.003189 0.003227	2.234 2.2735	32.34	0.00321	2.258	32.7	0.003168	2.213
	Kevlar	22.5	0.0144	4.629	22.5	0.0144	4.629	22.5	0.0144	4.629
	FG	22.5	0.0122	4.629	22.5	0.0122	4.629	22.5	0.0122	4.629
Mitchell	SS	28.5	0.008554	1.615	28.47	0.00857	1.618	28.41	0.008603	1.624
	Al-Alloy	32.27	0.00322	1.38	32.34	0.00321	1.377	32.5	0.003193	1.37
	Kevlar	22.5	0.0144	2.412	22.5	0.0144	2.412	22.5	0.0144	2.412
	FG	22.5	0.0122	2.412	22.5	0.0122	2.412	22.5	0.0122	2.412

## Conclusion and future work

The findings of this multi-objective optimization experimental-based study on wear resistance and wind turbine performance of blades made of stainless steels, Al-Alloy, Kevlar and fiber glass reinforced epoxy are presented in this paper. The erosion rate results are extracted from experimental work carried out in this research. Meanwhile, the performance indicator (lift-to-drag ratio) is extracted from two previous studies. The parameters investigated are impact angle, impact speed and sand particle size. The erosion rate and lift-to-drag ratio were evaluated, analyzed and optimized. Mathematical regression models for erosion rates were developed using MATLAB. Multi-objective optimization is carried out on these regression models. The main findings of this research are as follows:

1. The most affecting factor on the erosion rate of the wind turbine blades is the impact angle (angle of attack AoA). Furthermore, the impact angle is disproportional with the erosion rate in case of metallic materials, however, it is proportional with the erosion rate in the non-metallic materials cases.
2. The impact angle plays a great role in adjusting the performance coefficients of the wind turbine as the lift and drag coefficients. Keeping the impact angle under 45° helps in producing sound power out of the wind turbines.
3. The optimal solution results for the best adjustable impact angle of the blade in order to avoid the erosion wear and to improve the turbine productivity are obtained for each used material. In the case of Kevlar and fiber glass materials, the optimal solution converges to the lower boundary of the impact angle values at 22.5°.
4. Meanwhile, the trade-off between erosion rate and lift-to-drag ratio solutions for the stainless steel and Al-Alloy are obtained. For stainless steel, whether for Viterna or Mitchell model, the optimal impact angle ranges between 28° to 29° with a corresponding erosion rate of 0.0085 mg/g approximately. The optimal lift-to-drag ratio is different for Viterna and Mitchell as recording on average 2.85 and 1.62, respectively.
5. Similarly, the optimal impact angle for the Al-Alloy is ranging between 32.2° to 32.8° achieving an erosion rate of 0.0031 approximately. The optimal lift-to-drag ratios are 2.25 and 1.375 on average for Viterna and Mitchell, respectively.
6. The three muti-objective optimization algorithms; MOGA, MOPSA and WVGWO, used in this work showed similar optimal solutions, hence, no algorithm outperformed the other due to the single variable objective functions.
7. The findings of this study can guide material selection for wind turbine blades by providing insights into how different materials, both metallic and non-metallic, perform under varying operational conditions such as impact angle, impact speed and particle size. This study can help optimize blade design for improved erosion resistance and aerodynamic efficiency.
8. Remarkably, the study lacks in-depth microstructural analysis using SEM analysis and XRD in order to investigate the erosion mechanisms of metallic and non-metallic materials, therefore, it is highly recommended to add this investigation in future work for this study.
9. Finally, future research may focus on exploring advanced coatings or hybrid materials to further enhance erosion resistance, as well as examining the integration of these materials into real-world wind turbine designs for improved performance and lifetime.

## Data Availability

The data presented in this study are available on request from the corresponding author.
